# Industrial PLC Network Modeling and Parameter Identification Using Sensitivity Analysis and Mean Field Variational Inference

**DOI:** 10.3390/s23052416

**Published:** 2023-02-22

**Authors:** Raelynn Wonnacott, David S. Ching, John Chilleri, Cosmin Safta, Lee Rashkin, Thomas A. Reichardt

**Affiliations:** Sandia National Laboratories, 7011 East Avenue, Livermore, CA 94550, USA

**Keywords:** power line communications, mean field variational inference, sensitivity analysis, MIMO

## Abstract

A multiple input multiple output (MIMO) power line communication (PLC) model for industrial facilities was developed that uses the physics of a bottom-up model but can be calibrated like top-down models. The PLC model considers 4-conductor cables (three-phase conductors and a ground conductor) and has several load types, including motor loads. The model is calibrated to data using mean field variational inference with a sensitivity analysis to reduce the parameter space. The results show that the inference method can accurately identify many of the model parameters, and the model is accurate even when the network is modified.

## 1. Introduction

Power line communications (PLC) systems are becoming increasingly common and are a key component of smart grids, as they allow grid usage to be monitored without adding extensive infrastructure. Currently, PLC systems are commonly used for automatic meter reading and communication between electrical substations, but there is a wide range of potential applications, including dynamic load management, appliance and lighting control, device-specific billing, and power outage detection [1,2,3,4,5,6]. There are three classifications of PLC systems based on bandwidth: ultra narrowband (UNB) systems have frequencies ranging from 0.3–3 kHz, narrowband (NB) systems have a frequency range of 3–500 kHz, and broadband (BB) systems have frequencies of 1.8–250 MHz [7]. Lower transmission frequencies have slower data transmission rates but are less affected by losses and are, therefore, more effective over longer distances. In contrast, broadband systems are limited to applications within the home or industrial facilities but have much faster data transmission rates.

Power line networks are far from ideal for signal transmission. The loads, wires, junctions, and network configuration all affect the signal transmission, resulting in systems with high variability and noise. PLC systems must, therefore, be customized for individual systems to effectively transmit signals, which requires developing models that characterize transmission pathways for individual networks.

Two types of PLC models have been developed: top-down and bottom-up models [8,9]. The top-down approach generates a parametric model based on the transmission line (TL) theory [10] but does not incorporate information about the network topology and loads. The model parameters are found by fitting the model output to the data. The top-down modeling approach is best used when the full network information is unavailable, but measurements of the network are available. Because the network topology is not incorporated into the model, the model can no longer be used if the network components or the topology are changed.

The bottom-up model uses TL theory in combination with the network topology and conductive pathways to computing signal throughput [11]. Unlike top-down models, they can be easily modified if network components are changed. There are a number of previous works constructing bottom-up models for different applications [8,9,12,13,14,15]. The disadvantages of bottom-up models are that they require full knowledge of the network topology and pathways and that they cannot easily be modified to match measurements.

Most PLC models have been developed for home networks and incorporate either 2-conductor cables or 3-conductor cables [8,12,13,14,15,16,17,18,19,20,21,22,23,24,25,26], although several models have been extended to 4-conductor cables [27,28]. In this work, the target networks are industrial networks, which generally have 3-phase power throughout the facilities, so we model the network using 4-conductor cables (3-phase conductors and a ground conductor). Industrial facilities typically have a large number of motors present, unlike home networks, so we incorporate motor models in our network.

Our previous work (Ref. [29]) combined top-down and bottom-up models to gain the advantages of both. It used a Bayesian inference and sampling method called Transitional Markov Chain Monte Carlo (TMCMC) to calibrate model parameters to data. TMCMC effectively calibrates up to approximately 20 parameters, but it became computationally intractable to calibrate more, and a large PLC network model can contain many more unknown parameters. This work instead uses a Bayesian inference method called mean-field variational inference to calibrate model parameters and uses a more complex industrial network model instead of the home network model in Ref. [29]. Our efforts begin with the analysis of a single network, and then expand to multiple network realizations, then address the challenge of inferring load types. This paper is organized as follows: Section 2 describes the PLC network model for an industrial network, Section 3 describes mean-field variational inference, Section 4 contains results, and Section 5 contains conclusions and suggestions for future work.

## 2. PLC Network Model

The PLC network model used in this paper is a modified version of the networks used in Refs. [12,29], which are models of home networks. There are a number of differences between this work and Ref. [29]: this work models 4-conductor cables to account for three-phase wiring typical of industrial facilities instead of 2-conductor cables, the load models are different and account for motors, and the networks contain a larger number of separate circuits and loads.

### 2.1. Network Topology

**Figure 1:**
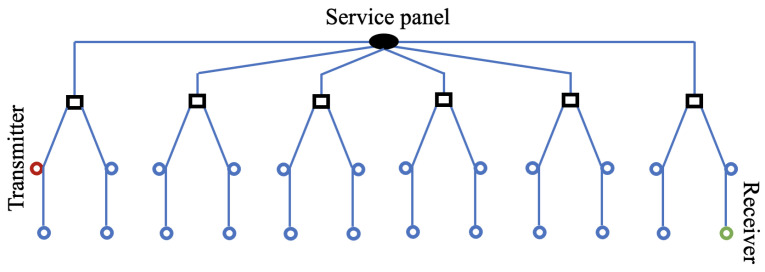
Network topology. Blue circles represent loads, and black squares are junction boxes.

The PLC network consists of a number of rooms, each on a separate circuit connected to the service panel, as shown in Figure 1. For this work, we use six rooms, each containing four outlets. Each circuit is connected to a junction box, from which cables leading to the outlets are connected. The outlets are organized in a bus connection, where only two cables are connected directly to the junction box. The service panel and junction boxes are assumed to be ideal connections with zero impedance. Two outlets in the facility are connected to the transmitter and receiver; the rest are connected to loads. The loads in the rooms that are not at wire terminations are connected in parallel to the wiring.

### 2.2. Cable Model

**Figure 2:**
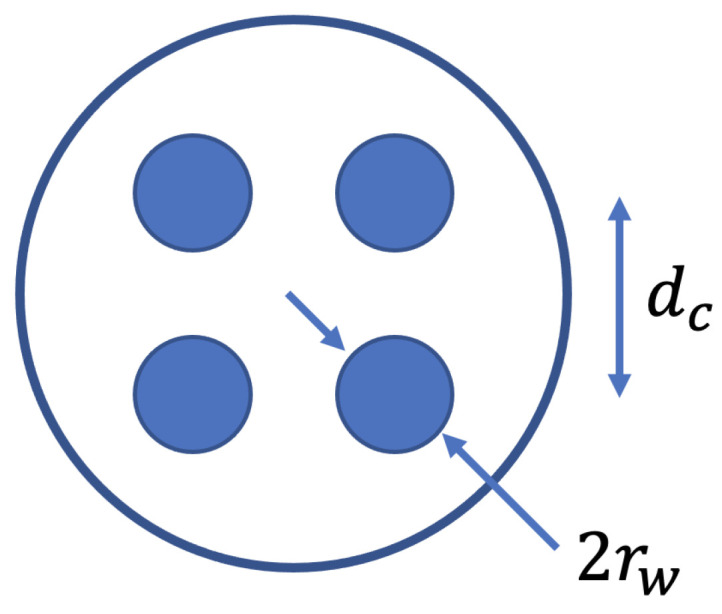
Schematic of the cable cross-section.

The cables are modeled using TL theory [10,30]. It is assumed that all four conductors in the cable have identical diameters and materials, and the conductors have a square layout, shown in Figure 2. The resistance, inductance, capacitance, and conductance are designated R, L, C, and G with values:

(1) $$\begin{array}{cc} R & =\left[\begin{array}{ccc} 2r & r & r\\ r & 2r & r\\ r & r & 2r \end{array}\right]\;with\;r=\left\{\begin{array}{cc} \frac{1}{\sigma \pi r_{w}^{2}}, & \mathrm{if}\;r_{w}\leq 2\delta \\ \frac{1}{2r_{w}}\sqrt{\frac{\mu_{0}f}{\pi \sigma }}, & \mathrm{if}\;r_{w}>2\delta \end{array}\right. \end{array}$$

(2) $$\begin{array}{cc} L & =\;\left[\begin{array}{ccc} \frac{\mu_{0}}{\pi }log\left(\frac{d_{1,0}}{r_{w}}\right) & \frac{\mu_{0}}{2\pi }log\left(\frac{d_{1,0}d_{2,0}}{d_{1,2}r_{w}}\right) & \frac{\mu_{0}}{2\pi }log\left(\frac{d_{1,0}d_{3,0}}{d_{1,3}r_{w}}\right)\\ \frac{\mu_{0}}{2\pi }log\left(\frac{d_{1,0}d_{2,0}}{d_{1,2}r_{w}}\right) & \frac{\mu_{0}}{\pi }log\left(\frac{d_{2,0}}{r_{w}}\right) & \frac{\mu_{0}}{2\pi }log\left(\frac{d_{2,0}d_{3,0}}{d_{2,3}r_{w}}\right)\\ \frac{\mu_{0}}{2\pi }log\left(\frac{d_{1,0}d_{3,0}}{d_{1,3}r_{w}}\right) & \frac{\mu_{0}}{2\pi }log\left(\frac{d_{2,0}d_{3,0}}{d_{2,3}r_{w}}\right) & \frac{\mu_{0}}{\pi }log\left(\frac{d_{3,0}}{r_{w}}\right) \end{array}\right] \end{array}$$

(3) $$\begin{array}{cc} C & =\mu_{0}\epsilon L^{-1} \end{array}$$

(4) $$\begin{array}{cc} G & =2\pi f\;tan(\delta_{L})\;C \end{array}$$

where $r_{w}$ is the conductor radius, $\mu_{0}=4\pi \;\cdot \;10^{-7}$ [H/m] is the vacuum permeability, $\sigma =5.8\;\cdot \;10^{7}$ [S/m] is the conductivity of copper, $\epsilon =3.19\;\cdot \;10^{-11}$ [F/m] is dielectric constant of PVC, *f* is the frequency. The loss tangent $tan(\delta_{L})$ is assumed to be zero, so G=0. The distances between conductors are $d_{0,1}=d_{0,3}=d_{1,2}=d_{2,3}=d_{c}$ and $d_{0,2}=d_{1,3}=\sqrt{2}d_{c}$, where $d_{c}$ is given by the relation:

(5) $$d_{c}=4\;\cdot \;10^{-4}+3.02r_{w}$$

This relation is from Ref. [29] and was found through a linear fit between conductor radius $r_{w}$ and conductor-to-conductor distance for Thermoplastic High Heat-resistant Nylon (THHN) wires between gauge sizes 6 and 16. The skin depth δ is given by

(6) $$\delta =\frac{1}{\sqrt{\pi \mu_{0}\sigma f}}$$

### 2.3. Load Models

**Figure 3:**
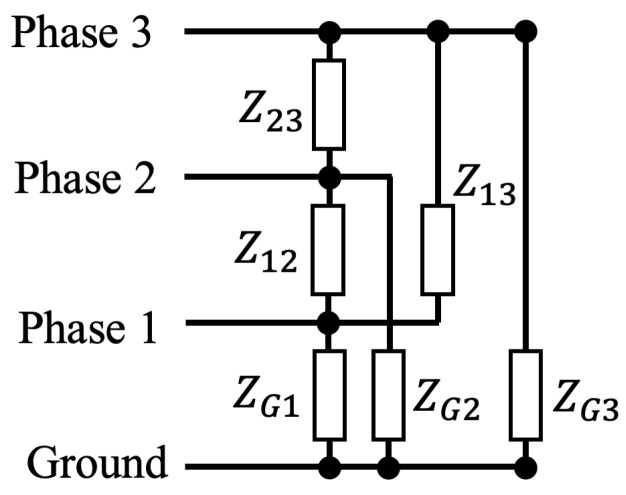
Schematic of load connections to wiring.

Each load consists of six component loads connecting each conductor in the three-phase cable with each other conductor, as shown in Figure 3. The components of each load connected to the ground cable are designated $Z_{G1}$, $Z_{G2}$, and $Z_{G3}$, and the three phase-to-phase components are designated $Z_{12}$, $Z_{13}$, and $Z_{23}$.

There are three types of loads: constant impedance, double RLC loads, and motor loads. Constant impedance loads are parameterized by a resistance $R_{const}$ and leakage capacitance $C_{c,leak}$. The three phase-to-phase components are equal to $R_{const}$, and the three ground-to-phase components are the impedance of the leakage capacitance. The load equations for a constant impedance load are

(7) $$\begin{array}{cc} Z_{12} & =Z_{13}=Z_{23}=R_{const} \end{array}$$

(8) $$\begin{array}{cc} Z_{G1} & =Z_{G2}=Z_{G3}=\frac{1}{j\omega C_{c,leak}} \end{array}$$

where ω=2πf. The constant impedance load is a model for simple loads such as incandescent lights and battery chargers.

**Figure 4:**
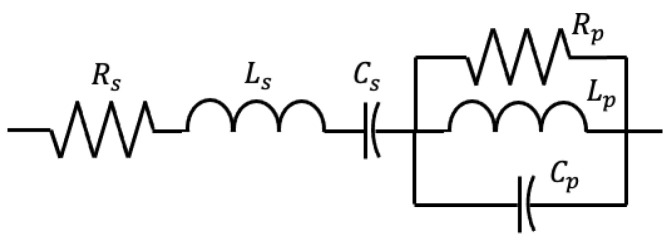
Double RLC phase-to-phase load circuit.

A double RLC load has a series RLC circuit connected to a parallel RLC circuit, as shown in Figure 4. Unlike the constant impedance load, there is an asymmetry in the phase-to-phase components. The double RLC load has 9 parameters, which are $R_{s}$, $\omega_{0s}$, $\zeta_{s}$, $R_{p}$, $\omega_{0p}$, $\zeta_{p}$, $\Delta_{1}$, $\Delta_{2}$, and $C_{d,leak}$. The load equations are

(9) $$\begin{array}{cc} Z_{12} & =\frac{2j\frac{\omega }{\omega_{0p}}R_{p}\zeta_{p}}{1+2j\frac{\omega }{\omega_{0p}}R_{p}\zeta_{p}-\frac{\omega^{2}}{\omega_{0p}^{2}}}+R_{s}+2jR_{s}\zeta_{s}\left(\frac{\omega }{\omega_{0s}}-\frac{\omega_{0s}}{\omega }\right) \end{array}$$

(10) $$\begin{array}{cc} Z_{13} & =Z_{12}\left(1+\Delta_{1}\right) \end{array}$$

(11) $$\begin{array}{cc} Z_{23} & =Z_{12}\left(1+\Delta_{2}\right) \end{array}$$

(12) $$\begin{array}{cc} Z_{G1} & =Z_{G2}=Z_{G3}=\frac{1}{j\omega C_{d,leak}} \end{array}$$

where the parameters $\Delta_{1}$ and $\Delta_{2}$ provide asymmetry in the load. $\Delta_{2}$ Note that each RLC circuit is converted from *R*, *L*, and *C* to natural frequency $\omega_{0}$ and damping factor ζ using the formula:

(13) $$\begin{array}{cc} \omega_{0} & =\frac{1}{\sqrt{LC}} \end{array}$$

(14) $$\begin{array}{cc} \zeta & =\frac{R}{2}\sqrt{\frac{C}{L}} \end{array}$$

The double RLC load can be considered a model for many loads with more complexity than constant impedance loads. For example, Refs. [31,32,33] took measurements of a variety of appliances and showed that many of them had resonant frequencies. Ref. [34] modeled a number of household appliances using combinations of parallel and series RLC loads, and the double RLC model is a generalization for many of the appliance models.

**Figure 5:**
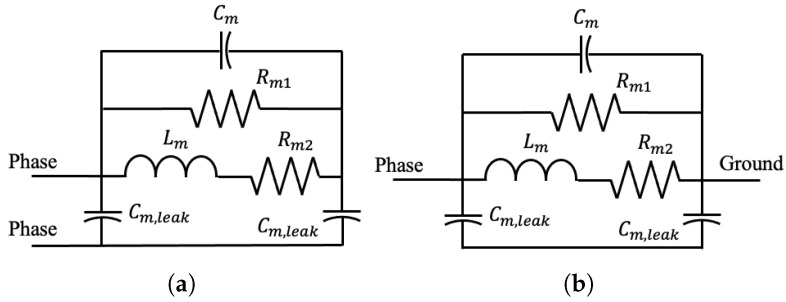
Motor model. (**a**) Phase-to-phase circuit. (**b**) Phase-to-ground circuit.

Industrial networks often contain large numbers of motors, so in this work, a separate model is used for motors [35]. This model is shown in Figure 5 and has been used in a variety of works, including Refs. [36,37,38,39,40] for three-phase induction motors.

The motor model has 5 parameters: $C_{m}$, $L_{m}$, $R_{m1}$, $R_{m2}$, and $C_{m,leak}$. However, the model is insensitive to $R_{m2}$ except at low frequencies, so its value is fixed at $R_{m2}=5\;\Omega$. The motor load formula can be reduced to

(15) $$\begin{array}{cc} Z_{12} & =\frac{1}{3}\frac{1}{j\omega \left(C_{m}+\frac{C_{m,leak}}{2}\right)+\frac{1}{R_{m1}}+\frac{1}{Z^{\prime }}} \end{array}$$

(16) $$\begin{array}{cc} Z_{13} & =Z_{23}=Z_{12} \end{array}$$

(17) $$\begin{array}{cc} Z_{G1} & =\frac{1}{3}\frac{1}{j\omega C_{m,leak}+\frac{1}{j\omega C_{m,leak}+\frac{R_{m1}Z^{\prime }}{j\omega C_{m}Z^{\prime }R_{m1}+Z^{\prime }+R_{m1}}}} \end{array}$$

(18) $$\begin{array}{cc} Z_{G2} & =Z_{G3}=Z_{G1} \end{array}$$

where $Z^{\prime }=j\omega L_{m}+R_{m2}$.

### 2.4. Network Generation

The cable lengths $l_{w}$ and conductor radius $r_{w}$ are generated with the parameter ranges in Table 1. The conductor radii for wires connected to the service panel are for wires with gauges between 12 and 6, while all other wires have ranges for gauges 14 to 10.

Each load in the network is generated with a random load type, where each load type has equal probability. The load parameters are generated over the ranges given in Table 1 and using either a uniform distribution or a log-uniform distribution.

When performing the inference, all of the parameters are allowed to vary over the ranges defined in Table 1 except cable length $l_{w}$ and conductor radius $r_{w}$. The conductor radius is fixed to the original value from when the parameters were assigned, and the cable length can vary ±1 m from its original value. This was done because it was found that performing inference when all the parameters were allowed to vary over large ranges gave poor results. The wire parameters are more likely to be known in a facility than the specific load parameters, so we chose to reduce the uncertainties associated with the wire parameters while keeping the load parameter ranges large.

### 2.5. Transmitter and Receiver Models

**Figure 6:**
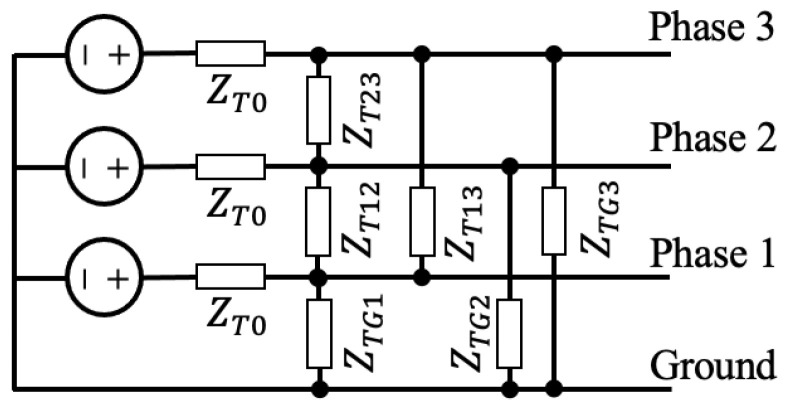
Transmitter model.

**Figure 7:**
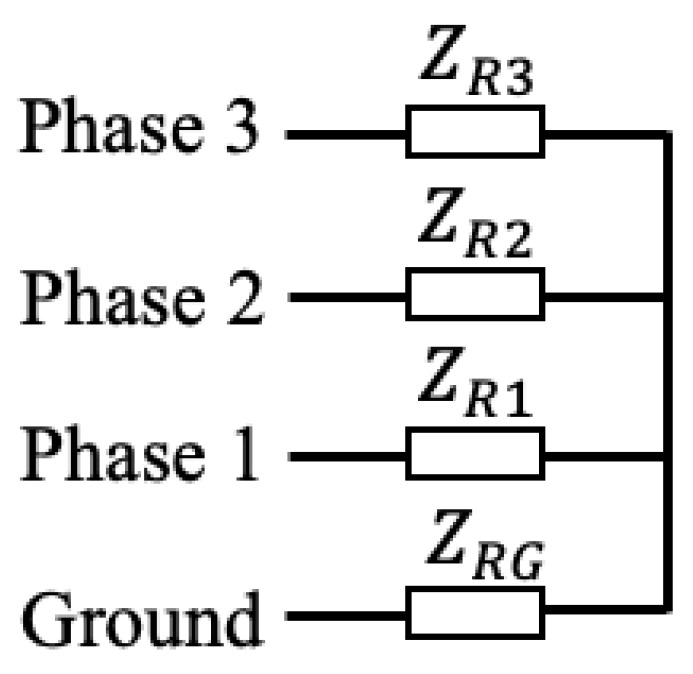
Receiver model.

The transmitter and receiver models are adapted from Ref. [20] for three-phase systems. The transmitter model is shown in Figure 6 and consists of one voltage source connected to each of the three-phase conductors. There is a single resistor between each pair of conductors as well as one after each voltage source. The receiver model is shown in Figure 7 and consists of a single resistor on each line.

The network is solved by reducing the entire network except for the transmitter and receiver to a single transmission parameter matrix, then computing the transfer function. The equations modeling the transfer function through the system are derived in the Appendix A.

### 2.6. Model Solver

**Figure 8:**
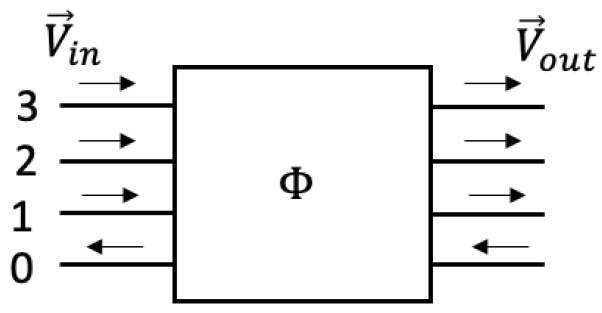
Transmission matrix definition.

The PLC network model is solved in Python using 4-port transmission parameter matrices. Each transmission line and load can be formulated as a 6×6 matrix, shown in Figure 8 and Equation (19).

(19) $$\left[\begin{array}{c} V_{1,in}\\ V_{2,in}\\ V_{3,in}\\ I_{1,in}\\ I_{2,in}\\ I_{3,in} \end{array}\right]=\Phi \left[\begin{array}{c} V_{1,out}\\ V_{2,out}\\ V_{3,out}\\ I_{1,out}\\ I_{2,out}\\ I_{3,out} \end{array}\right]$$

Each load can be put in the form of an admittance matrix $Y_{L}$ using the equation:

(20) $$Y_{L}=\left[\begin{array}{ccc} \frac{1}{Z_{G1}}+\frac{1}{Z_{12}}+\frac{1}{Z_{13}} & -\frac{1}{Z_{12}} & -\frac{1}{Z_{13}}\\ -\frac{1}{Z_{12}} & \frac{1}{Z_{G2}}+\frac{1}{Z_{12}}+\frac{1}{Z_{23}} & -\frac{1}{Z_{23}}\\ -\frac{1}{Z_{13}} & -\frac{1}{Z_{23}} & \frac{1}{Z_{G3}}+\frac{1}{Z_{13}}+\frac{1}{Z_{23}} \end{array}\right]$$

The admittance matrix of a load connected in parallel to transmission lines can be converted to a transmission parameter matrix using Equation (21), and the transmission parameter matrix for loads connected in series is given by Equation (22).

(21) $$\Phi_{parallelLoad}=\left[\begin{array}{cc} 1 & 0\\ -Y_{L} & 1 \end{array}\right]$$

(22) $$\Phi_{seriesLoad}=\left[\begin{array}{cc} 1 & -Z_{L}\\ 0 & 1 \end{array}\right]$$

where $Z_{L}=Y_{L}^{-1}$.

The network transfer function is computed over the frequency range [150 kHz, 30 MHz] for 200 discrete frequencies with logarithmic spacing. To obtain more simulated data, the resistances in the transmitter and receiver are varied, and the transfer function is computed for each combination of resistances, similarly to Ref. [29]. Table 2 and Table 3 show the resistance combinations used to compute transfer functions.

## 3. Mean Field Variational Inference

To estimate model parameters, we use a variational Bayes approach. We seek a posterior distribution that summarizes what we know about model parameters based on data and prior knowledge. In general, the posterior is calculated according to the Bayes rule.

(23) p(θ|d)∝p(d|θ)p(θ)

where $d\in R^{N_{d}}$ are the observed data, θ are the model parameters, and p() designates a probability. p(θ) is called the *prior probability*. The prior probability is based upon established knowledge in the area of a particular application. p(d|θ), the *likelihood*, measures agreement between model output and the simulated data for given parameter values. Hence, parameters that align with prior beliefs and result in a close match with the data have the highest posterior probability.

For this work, the prior probability is modeled with a uniform distribution where the bounds are set in Table 1. We define our likelihood using a Student’s t-distribution. Using a Student’s t-distribution resulted in faster convergence than a Gaussian function.

(24) $$p\left(d|\theta \right)=\prod_{i=0}^{N_{d}}T\left(d_{i}-y_{i},\sigma ,\nu \right)$$

where $y\in R^{N_{d}}$ represents our model output and T(μ,σ,ν) denotes a Student’s t-distribution with mean μ, variance σ, and degrees of freedom ν. For our purposes, we set σ and ν to be 0.4 and 1, respectively.

To apply Bayesian inference, p(θ|d) must be approximated as it can rarely be computed directly. Markov chain Monte Carlo (MCMC) methods are generally used to draw samples from the distribution of p(θ|d). In MCMC, samples are drawn using a Markov chain which is constructed to have stationary distribution p(θ|d). Unfortunately, when $N_{\theta }$ is large, MCMC methods often take a prohibitive amount of time, and convergence can be difficult to determine.

Variational inference methods provide an alternate approach to approximate otherwise intractable posterior distributions and are faster in most cases [41,42]. Assuming our posterior distribution comes from a specific family of distributions, we can cast the inference problem as an optimization problem. Variational inference methods most commonly use Kullback-Leibler divergence to measure the difference between distributions. We call a realization of our family of distributions $q_{\phi }\left(\theta \right)$.

Prior to performing inference, all network parameters are converted from the ranges shown in Table 1 to the range [0, 1] to ensure that they are all assigned equal weights. Accordingly, the variational family is chosen to be a Gaussian distribution truncated to be between 0 and 1. Here, we start with a standard Gaussian distribution and use rejection sampling to truncate the distribution according to our bounds. Parameter independence is the mean-field assumption, equivalent to setting the covariance matrix to the identity matrix. In general, the goal is to choose a variational family that is simple enough for efficient optimization but can capture a density close to p(θ,d).

Minimizing the KL divergence between our variational family and the posterior is equivalent to maximizing the Evidence Lower Bound (ELBO) with respect to $q_{\phi }\left(\theta \right)$.

(25) $$ELBO=E_{q_{\phi }\left(\theta \right)}\left[\log p\left(d,\theta \right)-\log q_{\phi }\left(\theta \right)\right]$$

For details on the derivation of the ELBO, see Ref. [41].

To maximize our objective function, it is desirable to have gradients of Equation (25). Finding an exact gradient is typically difficult as this would require gradients of both the model and variational distribution. We use a stochastic gradient estimator as we cannot compute gradients of arbitrarily complicated PLC models and variational distributions. To optimize the ELBO with stochastic optimization, we need an estimator of the gradient, which can be computed using samples from the variational distribution.

(26) $$E_{q_{\phi }\left(\theta \right)}\left[\nabla_{\phi }q_{\phi }\left(\theta \right)\left(\log p\left(d,\theta \right)-\log q_{\phi }\left(\theta \right)\right)\right]$$

This is called the score function gradient estimator, and its derivation can be found in Ref. [43]. As the derivative is taken with respect to the parameters of *q*, this approach does not require the calculation of the derivative of p(d,θ).

Due to the high variance of the score function estimator, using this alone often leads to poor results. It is common to use this estimator combined with various variance reduction techniques. Using a higher number of samples will lead to improved estimates of the gradient and faster convergence, although it also increases computational costs. In our work, we use a control variate strategy, where terms are added to the estimator that has zero expectation but is specifically chosen to reduce variance. Essentially, terms of the form $\log q_{\phi }\left(\theta \right)g\left(\theta \right)$ are replaced with $\log q_{\phi }\left(\theta \right)\left(g\left(\theta \right)-b\right)$ where *b* is a constant and *g* is not to be differentiated with respect to ϕ. The identity $E_{q_{\phi }\left(\theta \right)}\left[\nabla_{\phi }\left(\log q_{\phi }\left(\theta \right)\times b\right)\right]$ ensures this does not change the mean of our gradient estimator. A carefully chosen *b* can reduce the variance of our estimator. The value *b* is referred to as a baseline. In this work, our baseline is constructed using a decaying running average of samples from the objective function. For further discussion of baselines, see Ref. [44].

To perform inference on our model, we use the probabilistic software package called pyro [45,46]. The score function gradient estimator is computed using 14 samples, which are computed in parallel. An AdaGrad optimizer with an initial learning rate of 0.6 converges samples to the posterior distribution [47].

## 4. Results

Model calibration relies upon data simulated using the PLC model with randomly generated parameters to compute a transfer function for subsequent inference of network parameters. This also allows a direct comparison between the randomly generated parameters and the inferred parameters. 1.0 Decibel of noise is added to the transfer function before applying mean field variational inference.

Each network model can have over 100 parameters, and a parameter space this high-dimensional can make inferring parameters challenging. Furthermore, many of the network parameters are from cables or loads far from the main path between transmitter and receiver and have very little effect on signals between them. Therefore, this study uses sensitivity analysis to determine which parameters contribute least to the variance of the transfer function, similar to Ref. [29]. Computing a full sensitivity analysis as was done in Ref. [29] is computationally expensive, so this study instead uses an approximate sensitivity analysis.

To dramatically reduce the number of model evaluations that would otherwise be required by a global sensitivity analysis, a one-at-a-time (OAT) approach is employed. Varying each parameter ten times at values distributed throughout their domain while fixing all other parameters at their nominal values, the variation, defined as the two-norm of the difference with the nominal transfer function, is calculated with respect to each parameter. The total variation is defined as the sum of all of the variations due to each parameter. An estimate of the relative sensitivities of the parameters is obtained by normalizing each parameter’s variation by the total variation. Then, based on an adaptively selected threshold, parameters are kept or discarded based on their contribution. It was found through a series of tests that a one-half percent threshold often reduced the parameter space by more than half and, correspondingly, the runtime by a significant margin while achieving virtually equivalent results to that of the full parameter model.

### 4.1. Demonstration on Single Network

As an initial demonstration, a network is generated with random load types, but all the network parameters are assigned a “true” value of 0.25, so the reader can easily identify mispredicted parameters. This demonstration is to quantify the accuracy of the inference methodology so the loads are fixed to their true types.

**Figure 9:**
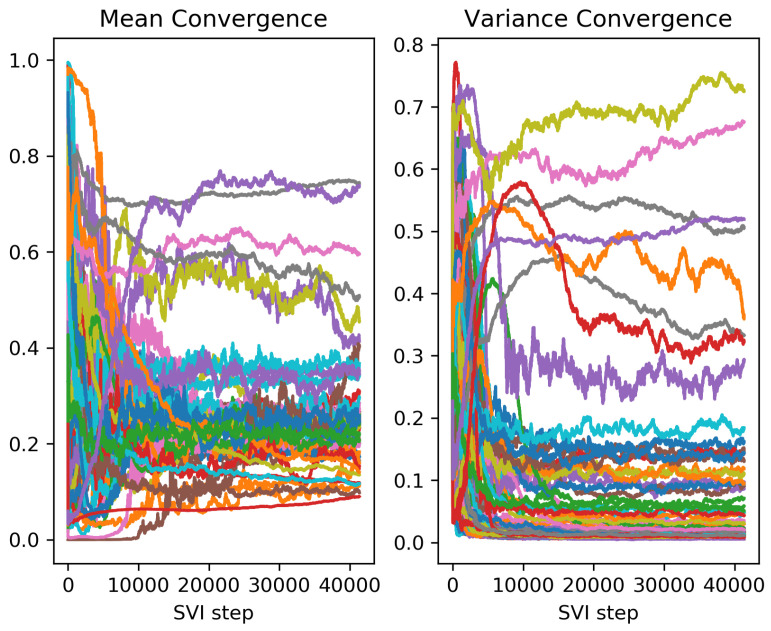
Mean value of parameters vs iteration (**left**) and variance vs iteration (**right**). Each line corresponds to a different parameter.

The mean and variance of each sensitive parameter are shown in each iteration step in Figure 9. The network has a total of 133 parameters, and 75 of them are sensitive parameters. All but 6 of them converged to mean values between 0.08 and 0.4, showing that mean field variational inference can accurately infer most of the sensitive parameters. Table 4 shows the mean parameter values at the end of the run ordered by decreasing sensitivity. The most sensitive parameters are inferred very accurately, while the accuracy noticeably decreases for less sensitive parameters.

**Figure 10:**
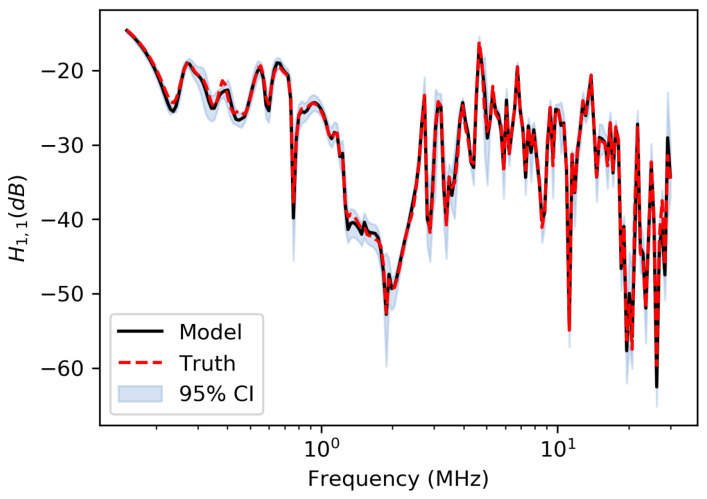
True transfer function (without added noise) and mean of transfer function computed from posterior with 95% confidence bounds.

Figure 10 shows the resulting transfer function component $H_{1,1}\left(f\right)$ along with the 95% confidence interval computed from the push-forward posterior. The push-forward posterior is the distribution of the transfer function computed from samples drawn from the posterior. The figure shows that the inference methodology accurately fits the model to the true transfer function, and the uncertainties are highest at frequencies where the transfer function has a peak.

The focus of this work is on the calibration methodology, so for brevity, we refrain from a detailed study of the full transfer function matrix. However, the transfer function was found to be full rank across the entire frequency range, justifying its usage for MIMO applications.

**Figure 11:**
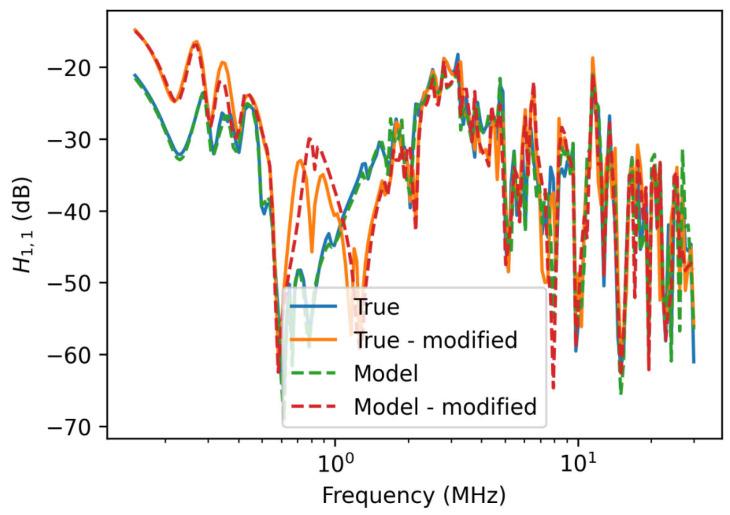
Transfer function from the original network and network modified by powering offloads in two rooms.

One of the main advantages of a bottom-up model is that the model can still be used if the network changes. Here, a new network is generated with random parameter values over the range [0, 1] and then calibrated with mean field variational inference. The network is then modified by powering off all of the the loads in the two central rooms of the network. This is done by assigning them as constant loads with the resistance of $R_{const}=10^{6}\;\Omega$ and parasitic capacitance $C_{c,leak}=0.1$ nF. Figure 11 shows the transfer function of the model with the loads turned off compared with the true model. While there are slight discrepancies, the calibrated model is very accurate despite not having been trained using data from the modified network.

### 4.2. Demonstration on Multiple Networks

**Figure 12:**
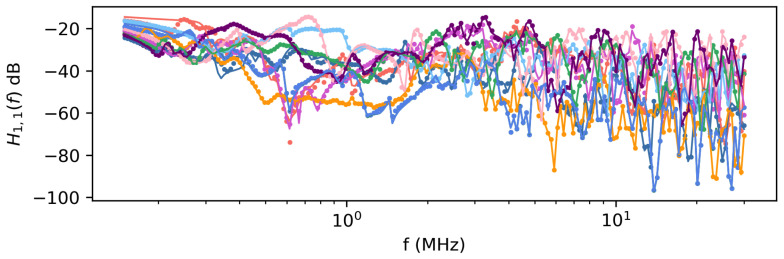
True transfer function component $H_{1,1}\left(f\right)$ and inferred transfer function for 10 different realizations of the network. Each color corresponds to a different realization. The dots are the true transfer function and lines are the inferred transfer function.

The results in Section 4.1 showed that the inference method could calibrate the model for a single realization of the network. Next, the method is repeated 10 times for different network realizations to demonstrate that the method is consistently able to calibrate the model. However, instead of setting the true values of the parameters to 0.25, all network parameters have randomly generated parameters over the range [0, 1]. Figure 12 shows the mean transfer function and true transfer function for each realization. Slight inaccuracies can be observed, particularly at frequency peaks, but generally, there is good agreement between the data and calibrated model.

Table 5 shows the magnitude of the average error for parameters in order of decreasing sensitivity. Each error in the table is averaged across the indicated parameters and the 10 realizations. The parameters with the highest sensitivity to the 10th highest sensitivity have an average error magnitude of 0.029, but the errors increase as the sensitivity decreases. Note that for parameters chosen randomly in [0, 1], randomly guessing the parameters would give an average error magnitude of 0.333. Since all the error magnitudes in Table 5 are significantly lower than 0.333, the inference method can consistently identify parameters, particularly for parameters with high sensitivity.

### 4.3. Inferring Load Types

A natural extension of this analysis is to consider the case where there are unknown load types that may be desirable to infer. In a home kitchen, for example, knowledge of a fridge and microwave may be known, while additional appliances may not be as clear, although a set of likely appliances may be assumed, given the setting. It follows that one may be interested in resolving the identity of loads; however, it must first be assessed whether this is a feasible task provided the selection of potential load types, the model to which they are applied, and the method of inferencing.

In the previous sections, the load types (constant, double RLC, or motors) during inference were identical to the true load types. In reality, it may not always be possible to know all of the loads in the networks, especially as loads may change during operation. It may, therefore, be important to infer load types in the network from data.

We performed an analysis to identify how much the transfer function changed based on load types. For brevity, we summarize the method and findings. Individual loads in a network were changed to a different load type, then inference was performed to see if a set of parameters could be found that resulted in an accurate transfer function even though one of the load types was wrong. It was found that if a motor load was replaced with a different load type or vice versa, it was generally impossible for the calibrated model to have an accurate transfer function. In contrast, constant loads and double RLC loads could generally be interchanged, and the calibrated model output would still be accurate. This suggests that constant and double RLC loads are too similar to identify from each other but that motor loads can be distinguished from the other load types.

We treat the constant and double RLC load types as a single group represented by the constant impedance load, as it has fewer parameters, and a slightly modified version of the algorithm offered in [29] is employed to predict load types. The algorithm initializes by assigning random load types to each load. This assignment is evaluated by performing a full parameter inference and recording the likelihood function. Then, three loads are randomly changed in type, and the change is accepted or rejected based on whether or not the subsequent full parameter inference yields better agreement than that of the previous assignment. This process continues for a predetermined number of steps, although it may be readily apparent to the user that changes are no longer being accepted, indicating potential convergence. The results are illustrated in Table 6.

The inference successfully identified all twenty-two of the unknown load groupings. While succeeding in this case, it should be noted that it may require a large number of iterations for the random triplets to happen upon the exact load identities. For this problem, with a limited number of iterations, it is more often the case that all but a random few will be inferred correctly. It should also be mentioned that positionally symmetric loads may interfere with the accuracy of load type inference. Positionally symmetric loads, in this context, are loads that are connected independently and identically to a network. As an example, two loads branching off of the same node by wires of equivalent length and matching properties are positionally symmetric loads. In particular, if the loads were to be swapped, the transfer function would be unaffected, as the network has effectively not changed. Consequently, in the presence of positionally symmetric loads, this method of load type inference would achieve equivalent objective function values for predicting the correct load types or the swapped, correct load types, which may be incorrect.

The success in our case is also attributable to the load types under consideration. When considering motor versus constant impedance loads, the impact of the two load types is substantially different, allowing for accurate resolution via tangible differences in the transfer function, even after inferring optimal load parameters. Additionally, accounting for only two load types simplifies the problem—more robust algorithms would likely be required if operating on a larger set of load types.

## 5. Conclusions

This work developed a PLC model for three-phase industrial networks and used mean field variational inference with sensitivity analysis to calibrate the model. The calibrated model has the advantages of both bottom-up and top-down models as it incorporates full knowledge of the network topology but can also be tuned to match data. The results showed that many of the parameters could be inferred accurately. When averaged across different network realizations, the 10 most sensitive parameters had an average error of 2.9% after calibration, and the 40 most sensitive parameters had an average error of 7.1%. This work also found good agreement between the transfer function produced by the calibrated model and a network modified by powering offloads, suggesting that the model maintains accuracy when the network undergoes changes such as loads being powered off.

This study used mean field variational inference for calibration, whereas our previous work (Ref. [29]) used transitional Markov chain Monte Carlo (TMCMC). The key difference between the methods is that mean field variational inference assumes the distribution of each parameter is independent of all other parameters and follows an assumed distribution profile, which in this case is Gaussian. While these assumptions allow mean field variational inference to converge to a solution faster than TMCMC and work better in high dimensional parameter spaces, care must be taken that it is only applied to problems where the assumptions are valid. Our previous work found that very few network parameters were strongly correlated, and generally, the distributions had a single mode. That, together with the accurate results obtained here, justifies using mean field variational inference.

Our analysis of inferring load types found that motor loads could be distinguished from constant or double RLC loads. In a 6-room network, all 22 motor loads were inferred correctly, but constant loads could not be distinguished from double RLC loads. This raises the question of what kinds of loads are needed for a PLC model. It is possible that a reasonable PLC model could contain one of those two types of loads rather than both.

The accuracy of the network structure and load models should be considered when analyzing PLC models. In this and many other previous works, both the network structure and load models were generated using intuition and generalizations rather than from statistics in actual applications. While this is done due to the lack of available statistics and does not discredit such works, it must be taken into account that the models may not reflect PLC networks in actual applications.

There are several directions future works could explore. One area where more research is needed is accurate load modeling. Most load models in PLC network models are unrealistic due to the lack of data from loads common in households and industrial networks. More work is needed to infer load types or cable parameters from data. Here and in Ref. [29], a greedy search algorithm is used, but that method is expensive and can be inaccurate. Future work could further explore the potential for MIMO applications in industrial facilities. Finally, research is needed to measure facilities and develop calibrated models custom to each facility.

## Figures and Tables

**Table 1:** Parameter ranges and distributions.

Parameter	Minimum	Maximum	Distribution Type
$r_{w}$ [mm] connected to service panel	1.03	2.06	uniform
$r_{w}$ [mm] in rooms	0.81	1.29	uniform
$l_{w}$ [m]	2	20	uniform
$R_{const}$ [Ω]	10	200	log-uniform
$C_{c,leak}$ [nF]	0.1	2.0	uniform
$R_{s}$ [Ω]	10	3000	log-uniform
$\omega_{0s}$ [Mrad/s]	0	30	uniform
$\zeta_{s}$	0.1	2	uniform
$R_{p}$ [Ω]	10	3000	log-uniform
$\omega_{0p}$ [Mrad/s]	0	30	uniform
$\zeta_{p}$	0.1	2	uniform
$\Delta_{1}$	−0.1	0.1	uniform
$\Delta_{2}$	−0.1	0.1	uniform
$C_{d,leak}$ [F]	0.1	2.0	uniform
$C_{m}$ [nF]	0.1	1.0	uniform
$L_{m}$ [mH]	5	20	uniform
$R_{m1}$ [Ω]	2000	15,000	uniform
$C_{m,leak}$ [nF]	0.2	5.0	uniform

**Table 2:** Transmitter resistance combinations.

Parameter	Transmitter 1	Transmitter 2
$Z_{T0}$	50Ω	100Ω
$Z_{TG1}$	50Ω	100Ω
$Z_{TG2}$	50Ω	100Ω
$Z_{TG3}$	50Ω	50Ω
$Z_{T12}$	100Ω	50Ω
$Z_{T13}$	100Ω	100Ω
$Z_{T23}$	100Ω	100Ω

**Table 3:** Receiver resistance combinations.

Parameter	Receiver 1	Receiver 2
$Z_{RG}$	50Ω	100Ω
$Z_{R1}$	50Ω	100Ω
$Z_{R2}$	50Ω	100Ω
$Z_{R3}$	50Ω	50Ω

**Table 4:** Mean parameter values from posterior in order of decreasing sensitivity, ordered left to right, then down. The true values are 0.25.

0.259	0.251	0.250	0.245	0.256	0.202
0.253	0.252	0.256	0.342	0.174	0.249
0.236	0.261	0.249	0.240	0.284	0.233
0.264	0.239	0.199	0.242	0.243	0.308
0.247	0.267	0.244	0.259	0.257	0.243
0.253	0.245	0.247	0.251	0.344	0.257
0.251	0.237	0.252	0.259	0.241	0.113
0.260	0.233	0.253	0.209	0.272	0.249
0.237	0.295	0.163	0.161	0.245	0.119
0.414	0.403	0.199	0.744	0.455	0.367
0.254	0.183	0.207	0.154	0.352	0.098
0.596	0.507	0.136	0.117	0.246	0.140

**Table 5:** Error magnitude of inferred parameters averaged across 10 realizations and the parameters in the given range. Parameters are ordered with decreasing sensitivity.

Parameters	Average Error Magnitude
1–10	0.029
10–20	0.080
20–40	0.088
40–60	0.176

**Table 6:** True and predicted load types. The load names are room number and outlet number. The * indicates that the constant impedance load was used as the representative member during the computations.

Load	True	Predicted
R1-O2	Constant	{Constant *, Double RLC}
R1-O3	Motor	Motor
R1-O4	Constant	{Constant *, Double RLC}
R2-O1	Motor	Motor
R2-O2	Motor	Motor
R2-O3	Motor	Motor
R2-O4	Motor	Motor
R3-O1	Constant	{Constant *, Double RLC}
R3-O2	Constant	{Constant *, Double RLC}
R3-O3	Constant	{Constant *, Double RLC}
R3-O4	Motor	Motor
R4-O1	Double RLC	{Constant *, Double RLC}
R4-O2	Motor	Motor
R4-O3	Motor	Motor
R4-O4	Double RLC	{Constant *, Double RLC}
R5-O1	Motor	Motor
R5-O2	Constant	{Constant *, Double RLC}
R5-O3	Double RLC	{Constant *, Double RLC}
R5-O4	Motor	Motor
R6-O1	Constant	{Constant *, Double RLC}
R6-O2	Motor	Motor
R6-O3	Motor	Motor

**Figure A1:**
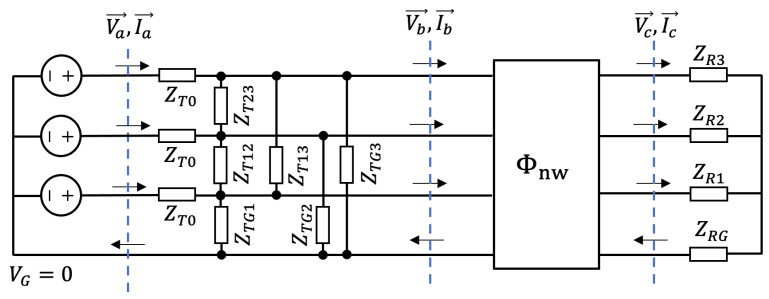
Schematic of the network reduced to a transmission parameter matrix.

## Data Availability

Data will be made available by the corresponding author upon reasonable request.

## Abbreviations

The following abbreviations are used in this manuscript:

BB	Broadband
ELBO	Evidence lower bound
MIMO	Multiple input multiple output
NB	Narrowband
PLC	Power line communications
RLC	Resistor-inductor-capacitor
TMCMC	Transisional Markov chain Monte Carlo
TL	Transmission line
UNB	Ultra narrowband

## Appendix A. Transfer Function Computation

A schematic of the network with the transmitter and receiver is shown in Figure A1, where the entire network except for the transmitter and receiver is reduced to a single transmission parameter matrix $\Phi_{\mathrm{nw}}$. Three locations *a*, *b*, and *c* are designated by the blue dashed lines. The transfer function is defined as

(A1) $${\vec{V}}_{c}=H{\vec{V}}_{a}$$

We define transfer functions through a part of the network as

(A2) $$\begin{array}{cc} {\vec{V}}_{b} & =H_{\mathrm{trans}}{\vec{V}}_{a} \end{array}$$

(A3) $$\begin{array}{cc} {\vec{V}}_{c} & =H_{\mathrm{nw}}{\vec{V}}_{b} \end{array}$$

Using the chain rule,

(A4) $$H=H_{\mathrm{nw}}H_{\mathrm{trans}}$$

We first compute $H_{\mathrm{nw}}$ by expanding the network parameter matrix $\Phi_{nw}$ into submatrices and then substituting in the receiver load:

(A5) $$\left[\begin{array}{c} \vec{V_{c}}\\ \vec{I_{c}} \end{array}\right]=\left[\begin{array}{cc} \phi_{nw,11} & \phi_{nw,12}\\ \phi_{nw,21} & \phi_{nw,22} \end{array}\right]\left[\begin{array}{c} \vec{V_{b}}\\ \vec{I_{b}} \end{array}\right]$$

We can also define submatrices of the inverse matrix

(A6) $$\left[\begin{array}{c} \vec{V_{b}}\\ \vec{I_{b}} \end{array}\right]=\left[\begin{array}{cc} \phi_{nw,11}^{-1} & \phi_{nw,12}^{-1}\\ \phi_{nw,21}^{-1} & \phi_{nw,22}^{-1} \end{array}\right]\left[\begin{array}{c} \vec{V_{c}}\\ \vec{I_{c}} \end{array}\right]$$

### Appendix A.1. Receiver Load

The receiver load matrix is defined as $Z_{\mathrm{rec}}$ where,

(A7) $$\vec{V_{c}}=Z_{\mathrm{rec}}\vec{I_{c}}$$

We assume that the power grid has a fixed voltage and, therefore, does not carry signals from the transmitter. For the particular receiver in Figure A1, the transmitter load matrix is computed as follows:

(A8) $$\begin{array}{c} \begin{array}{cc} \; & V_{c,1}=Z_{R1}I_{c,1}+\left(I_{c,1}+I_{c,2}+I_{c,3}\right)Z_{RG}\\ \; & V_{c,2}=Z_{R2}I_{c,1}+\left(I_{c,1}+I_{c,2}+I_{c,3}\right)Z_{RG}\\ \; & V_{c,3}=Z_{R3}I_{c,1}+\left(I_{c,1}+I_{c,2}+I_{c,3}\right)Z_{RG} \end{array} \end{array}$$

(A9) $$Z_{\mathrm{rec}}=\left[\begin{array}{ccc} Z_{RG}+Z_{R1} & Z_{RG} & Z_{RG}\\ Z_{RG} & Z_{RG}+Z_{R2} & Z_{RG}\\ Z_{RG} & Z_{RG} & Z_{RG}+Z_{R3} \end{array}\right]$$

Substituting Equation (A7) into the first row of Equation (A6) yields:

(A10) $$\vec{V_{b}}=\phi_{nw,11}^{-1}\vec{V_{c}}+\phi_{nw,12}^{-1}Z_{\mathrm{rec}}^{-1}\vec{V_{c}}$$

Reorganizing gives:

(A11) $$H_{\mathrm{nw}}={\left(\phi_{nw,11}^{-1}+\phi_{nw,12}^{-1}Z_{\mathrm{rec}}^{-1}\right)}^{-1}$$

It is also useful to compute an effective admittance $Y_{\mathrm{nw}}$ of the network and receiver. Equation (A7) can be substituted into Equation (A5) to obtain:

(A12) $$Y_{\mathrm{nw}}\vec{V_{b}}=\vec{I_{b}}$$

where:

(A13) $$Y_{\mathrm{nw}}=-{\left(\phi_{nw,12}-Z_{\mathrm{rec}}\phi_{nw,22}\right)}^{-1}\left(\phi_{nw,11}-Z_{\mathrm{rec}}\phi_{nw21}\right)$$

### Appendix A.2. Transmitter Computation

Three equations can be formed by applying Krichoff’s current law to the three conductors in the transmitter:

(A14) $$\begin{array}{c} \begin{array}{cc} I_{b1}= & I_{a1}-\frac{V_{b1}}{Z_{TG1}}-\frac{V_{b1}-V_{b2}}{Z_{T12}}-\frac{V_{b1}-V_{b3}}{Z_{T13}}\\ = & I_{a1}-\frac{Z_{T12}Z_{T13}+Z_{TG1}Z_{T13}+Z_{TG1}Z_{T12}}{Z_{TG1}Z_{T12}Z_{T13}}V_{b1}+\frac{1}{Z_{T12}}V_{b2}+\frac{1}{Z_{T13}}V_{b3}\\ I_{b2}= & I_{a2}-\frac{V_{b2}}{Z_{TG2}}-\frac{V_{a2}-V_{a1}}{Z_{T12}}-\frac{V_{b2}-V_{b3}}{Z_{T23}}\\ = & I_{a2}+\frac{1}{Z_{T12}}V_{b1}-\frac{Z_{TG2}Z_{T12}+Z_{TG2}Z_{T23}+Z_{T12}Z_{T23}}{Z_{TG2}Z_{T12}Z_{T23}}V_{b2}+\frac{1}{Z_{T23}}V_{b3}\\ I_{b3}= & I_{a3}-\frac{V_{b3}}{Z_{TG3}}-\frac{V_{b3}-V_{b1}}{Z_{T13}}-\frac{V_{b3}-V_{b2}}{Z_{T23}}\\ = & I_{a3}+\frac{1}{Z_{T13}}V_{b1}+\frac{1}{Z_{T23}}V_{b2}-\frac{Z_{TG3}Z_{T13}+Z_{TG3}Z_{T23}+Z_{T13}Z_{T23}}{Z_{TG3}Z_{T13}Z_{T23}}V_{b3} \end{array} \end{array}$$

The impedance law across resistors $Z_{T0}$ is

(A15) $$\frac{\vec{V_{a}}-\vec{V_{b}}}{Z_{T0}}=\vec{I_{a}}$$

Equations (A15) and (A12) are substituted into (A14) to eliminate the variables $\vec{I_{a}}$ and $\vec{I_{b}}$.

(A16) $$\begin{array}{c} \begin{array}{cc} Y_{nw,11}Y_{b1}+Y_{nw,12}Y_{b2}+Y_{nw,13}Y_{b3}= & \frac{V_{a1}-V_{b1}}{Z_{T0}}-Y_{T\alpha }V_{b1}+\frac{1}{Z_{T12}}V_{b2}+\frac{1}{Z_{T13}}V_{b3}\\ Y_{nw,21}Y_{b1}+Y_{nw,22}Y_{b2}+Y_{nw,23}Y_{b3}= & \frac{V_{a2}-V_{b2}}{Z_{T0}}-\frac{1}{Z_{T12}}V_{b1}-Y_{T\beta }V_{b2}+\frac{1}{Z_{T23}}V_{b3}\\ Y_{nw,31}Y_{b1}+Y_{nw,32}Y_{b2}+Y_{nw,33}Y_{b3}= & \frac{V_{a3}-V_{b3}}{Z_{T0}}+\frac{1}{Z_{T13}}V_{b1}+\frac{1}{Z_{T23}}V_{b2}-Y_{T\gamma }V_{b3} \end{array} \end{array}$$

where

(A17) $$\begin{array}{c} \begin{array}{cc} Y_{T\alpha } & =\frac{Z_{T12}Z_{T13}+Z_{TG1}Z_{T13}+Z_{TG1}Z_{T12}}{Z_{TG1}Z_{T12}Z_{T13}}\\ Y_{T\beta } & =\frac{Z_{TG2}Z_{T12}+Z_{TG2}Z_{T23}+Z_{T12}Z_{T23}}{Z_{TG2}Z_{T12}Z_{T23}}\\ Y_{T\gamma } & =\frac{Z_{TG3}Z_{T13}+Z_{TG3}Z_{T23}+Z_{T13}Z_{T23}}{Z_{TG3}Z_{T13}Z_{T23}} \end{array} \end{array}$$

This can be reordered to

(A18) $$\left[\begin{array}{ccc} 1+Z_{T0}Y_{nw,11}+Z_{T0}Y_{T\alpha } & Z_{T0}Y_{nw,12}-\frac{Z_{T0}}{Z_{T12}} & Z_{T0}Y_{nw,13}-\frac{Z_{T0}}{Z_{T13}}\\ Z_{T0}Y_{nw,21}-\frac{Z_{T0}}{Z_{T21}} & 1+Z_{T0}Y_{nw,22}+Z_{T0}Y_{T\beta } & Z_{T0}Y_{nw,23}-\frac{Z_{T0}}{Z_{T23}}\\ Z_{T0}Y_{nw,31}-\frac{Z_{T0}}{Z_{T31}} & Z_{T0}Y_{nw,32}-\frac{Z_{T0}}{Z_{T32}} & 1+Z_{T0}Y_{nw,33}+Z_{T0}Y_{T\gamma } \end{array}\right]\vec{V_{b}}=\vec{V_{a}}$$

By comparing to Equation (A2), it is evident that the matrix in Equation (A18) is the inverse of $H_{\mathrm{trans}}$. The transfer function is then computed using the chain rule in Equation (A4) with Equation (A11) and Equation (A18).
